# Impact of Wheat/Faba Bean Mixed Cropping or Rotation Systems on Soil Microbial Functionalities

**DOI:** 10.3389/fpls.2016.01364

**Published:** 2016-09-15

**Authors:** Sanâa Wahbi, Yves Prin, Jean Thioulouse, Hervé Sanguin, Ezékiel Baudoin, Tasnime Maghraoui, Khalid Oufdou, Christine Le Roux, Antoine Galiana, Mohamed Hafidi, Robin Duponnois

**Affiliations:** ^1^IRD, UMR LSTMMontpellier, France; ^2^Laboratoire Ecologie et Environnement (Unité Associée au CNRST, URAC 32), Faculté des Sciences Semlalia, Université Cadi AyyadMarrakech, Morocco; ^3^CIRAD, UMR LSTMMontpellier, France; ^4^Université Lyon 1, CNRS, UMR5558, Laboratoire de Biométrie et Biologie EvolutiveVilleurbanne, France; ^5^Laboratoire de Biologie et de Biotechnologie des Microorganisms, Faculté des Sciences Semlalia, Université Cadi AyyadMarrakech, Morocco

**Keywords:** arbuscular mycorrhizal fungi, cropping systems, nutrient uptake efficiency, microbial soil functions, Mediterranean region

## Abstract

Cropping systems based on carefully designed species mixtures reveal many potential advantages in terms of enhancing crop productivity, reducing pest and diseases, and enhancing ecological services. Associating cereals and legume production either through intercropping or rotations might be a relevant strategy of producing both type of culture, while benefiting from combined nitrogen fixed by the legume through its symbiotic association with nitrogen-fixing bacteria, and from a better use of P and water through mycorrhizal associations. These practices also participate to the diversification of agricultural productions, enabling to secure the regularity of income returns across the seasonal and climatic uncertainties. In this context, we designed a field experiment aiming to estimate the 2 years impact of these practices on wheat yield and on soil microbial activities as estimated through Substrate Induced Respiration method and mycorrhizal soil infectivity (MSI) measurement. It is expected that understanding soil microbial functionalities in response to these agricultural practices might allows to target the best type of combination, in regard to crop productivity. We found that the tested cropping systems largely impacted soil microbial functionalities and MSI. Intercropping gave better results in terms of crop productivity than the rotation practice after two cropping seasons. Benefits resulting from intercrop should be highly linked with changes recorded on soil microbial functionalities.

## Introduction

The ecological key processes that warrant the productivity and stability of terrestrial ecosystems have been designated as efficient models for sustainable agricultural management. Since it is well known that the functioning and stability of terrestrial ecosystems are dependent to plant biodiversity and species composition (Naeem et al., 1994; Tilman and Downing, 1994; Tilman et al., 1996; Hooper and Vitousek, 1997), mixing plant species in cropping systems reveals many potential advantages under various conditions to contribute to modern and sustainable agriculture (Vandermeer, 1989). In agroecosystems, multispecies cropping systems may (i) sustain biomass production and decrease the risk of crop failure in unpredictable environments, (ii) rehabilitate disturbed ecosystem services (i.e., water and nutrient cycling), and (iii) decrease risks of invasion, pests, and diseases through enhanced biological control or direct control of pests (Gurr et al., 2003). These cultural practices have often been considered as a practical application of ecological principles related on biodiversity, plant interactions, and other natural regulation mechanisms (Malézieux et al., 2009). Numerous different multispecies cropping systems can be designed by including various criteria such the frequency of land-use rotation, the intensity of intercropping, etc. (Garcia-Barrios, 2003). For instance, the intercropping, defined as the growth of more than one crop species or cultivar simultaneously in the same field during the same growing season (Ofori and Stern, 1987; Hauggaard-Nielsen et al., 2007), enhanced the use efficiency of environmental sources for plant growth resulting in stable yields (Hauggaard-Nielsen et al., 2001; Corre-Hellou and Crozat, 2005; Jensen et al., 2015). It has been also reported that the association of cereals and legumes at the same space and time led to higher yields and improved N (*via* biological N_2_ fixation for the legume) and P nutrition (Li et al., 2005; Betencourt et al., 2012; Latati et al., 2013, 2014).

The positive effects of species diversity in intercropping systems result from two main processes: complementarity and facilitation (Fridley, 2001; Hinsinger et al., 2011) whereas in rotation systems (i.e., legumes/cereals rotation), they occur through indirect feedback interactions (Schnitzer et al., 2011). It has been reported that these biological processes were mainly driven by soil microbe activities (Klironomos, 2002; Tang et al., 2014). For instance, the enhancement of P acquisition in the context of cereal/legume intercrops could occur as a consequence of microbial mediated processes involving soil fungi and bacteria (Wang et al., 2007). Positive feedback often involves changes in abundance and diversity of symbiotic mutualists such as nitrogen-fixing rhizobacteria and mycorrhizal fungi (van der putten et al., 2013). These biological processes have been particularly studied for their impacts on primary production, nutrient retention, and resilience after stress in natural prairie ecosystems (Tilman et al., 1996, 1997) or in natural forest ecosystems (Altieri, 1999). In contrast, few studies have been conducted in agricultural systems to determine the impacts of multispecies cropping systems on microbial soil functionalities and mycorrhizal soil infectivity in relation with crop productivity.

It has been suggested that Faba bean could enable diversification of the agrosystems (Garofalo et al., 2009; Köpke and Nemecek, 2010) and it has been highlighted the importance of the N and P contributed by faba bean in intercropping and rotation systems (Li et al., 2003). However, the Faba bean impacts on soil microbial functionalities in field conditions have been less investigated (Köpke and Nemecek, 2010). In the present study, two cereal/legume systems (intercropping and rotation) were investigated in a field experiment during two growing seasons. The specific aims of this study were (i) to evaluate the agronomic performance of wheat/Faba bean in intercrop and in rotation and (ii) to monitor the impacts of these cultural practices on the mycorrhizal soil infectivity and on the microbial soil functionalities.

## Materials and Methods

### Field Conditions and Experimental Design

The field experiment was performed in the 2011–2013 growing seasons in the Haouz plain at about 30 km at the East of Marrakech (31°4′60″ N and 7°3′0″ W, Morocco). Soil chemical properties (0–0.10 m layer) were as follows: pH (H_2_O) 7.2; carbon (%) 1.54; nitrogen (%) 0.08, C/N 19.2; Total P (mg.kg^-1^) 502.9 and Olsen P (mg.kg^-1^) 22.1. This soil exhibited a high content of available P presumably non-limiting for plant growth and resulting from large-P fertilizer applications during the last decades, until this part of the field was dedicated to organic farming 1 year before the beginning of this experiment. The regional climate of the experimental site is typical Mediterranean with surface soils regularly undergoing drying-rewetting cycles from the irregular distribution of rainfall. The annual average rainfall was 282 mm, mostly in Autumn/Winter (59%) and in Spring (22%). The dry season is from May to September. The mean air temperature is 17.9°C in autumn, 12.8°C in winter, 18.5°C in spring, and 24.7°C in summer. Soil, cropped in the previous growing season with durum wheat (*Triticum durum* Desf.), was plowed to a depth of 0.30 m in Summer and then shallowly harrowed to control weeds. No herbicide nor chemical fertilizers were applied. The experimental design had a randomized block design with two factors and four replication blocks. The factor was the cropping system (wheat/faba bean intercropping, rotation, or wheat monoculture). Plots were 3.0 m × 3.0 m; each main plot was spaced 1.0 m out from the next. The crops were sown in December 2011 and 2012 at a rate of 400 viable seeds m^-2^ in rows 0.18 m apart for wheat and at a rate of 200 kg.ha^-1^for faba bean. When intercropped, the two species were sown in the same row in order to maximize root proximity and plant-plant interactions. The experimental plot consisted of four rows 3 m long. Weeds were controlled by hand during the experiment. Hence, 3 treatments were examined, namely durum wheat as sole crop (W) and two cropping systems: durum wheat/Faba bean intercrop (WF) and wheat/Faba bean rotation (F+W).

### Plant Analyses

Wheat was considered as the main crop and faba bean as an intercrop component. Hence, the expected benefits of the cultural practices on yield productivity were only examined on wheat plants. After one and two growing seasons, at wheat tillering and at the same time, the total number of wheat plant and spike per plot were counted. Then 10 randomly chosen plants in the middle of the plot were harvested. The seeds from each plant were collected, counted, and weighed to determine the dry weight of 1000 seeds (grain yield). These measurements recorded on a plot basis were converted to hectare for statistical analysis. The aerial parts of each plant were then oven dried at 70°C during 2 weeks and weighed. After drying, shoot tissues were ground, ashed (500°C), digested in 2 ml HCl 6N and 10 ml HNO_3_ N for nitrogen and then analyzed by colorimetry for phosphorus (John, 1970). For nitrogen determination (Kjeldahl method), they were digested in 15 ml H_2_SO_4_ (36 N) containing 50 g l^-1^ of salicylic acid. Roots from five other randomly chosen plants in the middle of each plot were sampled and root subsamples of about 3 g each were taken. Each subsample was stained with 0.05% trypan blue in lactic acid according to Phillips and Hayman (1970); root colonization by AMF was then measured with the grid intersect method according to Giovannetti and Mosse (1980).

### Soil Microbial Analysis

After one and two growing seasons, soil cores (1 kg) were collected at 0- to 20-cm depth in each plot. About 10 soil samples were taken from each plot and pooled together. Soil samples were crushed and passed through a 2-mm sieve. Then hyphal length that is considered as a main component of the mycorrhizal soil infectivity (Kisa et al., 2007) was measured by the filtration-grid-line method on membrane filters according to Jakobsen and Rosendahl (1990). Patterns of *in situ* catabolic potential (ISCP) were designed to assess the functions of soil microbial communities and the microbial functional diversity in soil treatments after one and two growing seasons. The diversity of the catabolic potentials of the total soil bacterial community was evaluated according to Campbell et al. (2003) by a microrespirometry method performed in 96-well microtiter plates. In order to ensure the resumption of microbial activity, sterile distilled water was added to reach 30% of the water-holding capacity and plates were incubated 3 days in the dark at 28°C. Then soil wells received 28 organic substrate solutions (three wells per substrate). Stock solutions for thirteen carbohydrates (D-mannose, D-mannitol, D-trehalose, L-arabinose, D-xylose, D-sucrose, D-galactose, meso-inositol, D-sorbitol, L-rhamnose, L-arabitol, mesoerythriol, D-Glucose), eight carboxylic acids (citric acid, maleic acid, D,L-malic acid, oxalic acid, Na-gluconate, α-ketoglutaric acid, L-ascorbic acid) and seven amino acids (L-asparagine, D,L-valine, L-methionine, L-glutamine, D,L-alanine, D,L-serine, *N*-acetyl-D-Glucosamine) were prepared with distilled water and their concentrations were calculated to lead, respectively, 0.03, 0.04, and 0.004 mmol g^-^1 soil. Basal respiratory activity was calculated in triplicate with distilled water. The colorimetric detection plates were assembled and used according to MicroResp^TM^ (Aberdeen, UK) recommendations. Absorbance was measured at 572 nm with a Tecan infinite M200 reader before substrate spiking (t0) and after 6 h of incubation at 28°C (t6). For each well, absolute respiratory activity was calculated by subtracting the absorbance value at t0 from the value at t6. The average basal respiration value was then subtracted from all the individual substrate respiration values. For each carbon source, this substrate-specific respiratory activity was averaged and the value was finally divided by the sum of all the mean substrate-specific respiratory activities (pi value). The catabolic evenness (E) was calculated to determine the catabolic diversity of soil treatments. It represents the variability of catabolized substrates amongst the range of the targeted substrates and is calculated using the Simpson–Yule index E = 1/Σp2_i_ with p_i_ = (respiration response to individual substrates)/(Total respiration activity induced by all substrates for a soil treatment; Magurran, 1988). The catabolic evenness could be used to evaluate the ability of microbial communities to resist against environmental stress or disturbance (Magurran, 1988). Data were calculated for the individual responses to substrates but also for the average responses with carbohydrates, carboxylic acids, and amino acids.

### Statistical Analysis

All the data were subjected to a two-way analysis of variance and comparisons among means were made using the Newman–Keuls test (*P* < 0.05). The percentages of the mycorrhizal colonization were transformed by arcsin(sqrt) before the statistical analysis. The relationships between ISCP profiles table (2012 data) and yield variables table (2013 data) were analyzed using Between-Group CO-Inertia Analysis (BGCOIA). BGCOIA is a Co-Inertia Analysis on the two tables of group means obtained after a Between-Group Analysis (BGA, Thioulouse et al., 2012). As a first step, a BGA is therefore computed on the two data sets, considering each treatment as a group. The technical details of BGCOIA are given in Franquet et al. (1995). Examples of use and a comparison with other methods are presented in Thioulouse (2011) in the framework of k-tables data analysis methods. Let g be the number of groups (treatments here). The table of group means for SIR profiles is obtained by computing the means of each substrate within each treatment. This gives a new table, with g rows and p columns (p substrates). The same computations are done for the yield data table, leading to a second new table with g rows and q columns (q yield variables). A Co-Inertia Analysis is then performed on these two new tables. The rows of the initial tables can be projected into this analysis to help interpret the results (Lebart et al., 1984). Computations and graphical displays can be produced with the ade4 package for the R software (Thioulouse and Dray, 2007).

## Results

### Grain Yield and Biomass Production

After one cropping season, no significant effect on the wheat development has been recorded between the wheat monoculture and the wheat/faba bean intercropping (**Table 1**). After the second cropping season, crop data evidenced that total biomass yield, spike number, spike dry weight were higher in the intercropping treatment (WF) compared to the wheat monoculture (W) and Faba bean/wheat rotation (W+F) treatments with some enhancements resulting from the intercropping vs. monoculture of +84.2, +24.8, and 122.7%, respectively (**Table 1**). The thousand-seed weight was significantly higher in the WF and W+F treatments compared to the monoculture (W; **Table 1**). After the second growing season, the shoot P content was significantly increased in the intercropping and rotation treatments whereas nitrogen enhancement was only recorded in the WF treatment with (**Table 1**).

**Table 1:** Effects of cropping systems (W: wheat monoculture; WF: wheat/Faba bean intercropping; W+F: Wheat/Faba bean rotation) on total biomass yield (kg.ha^-1^), spike number per ha, spike dry weight (kg.ha^-1^), thousand-seed weight (TSW), mineral nutrition, mycorrhizal colonization of durum wheat and on soil catabolic evenness, and standardized average substrate-induced respiration (SIR) responses with each substrate group (carboxylic acids, amino-acids, and carbohydrates) in 2012 and 2013 in the field experiment located in the Haouz plain at about 30 km at the East of Marrakech (Morocco).

	Treatments					
	2012			2013		
	W	WF	W+F	W	WF	W+F
Total biomass yield (kg.ha^-1^)	4662 (78.5)^1^ a^2^	3897 (49.3) a	–	4434 (66.1) a	8167 (97.3) b	5038 (94.6) a
Spike number per ha (× 10^4^)	219.3 (12.2) a	213.7 (24.4) a	–	192.3 (37.5) a	240.1 (10.2) b	179.1 (39.9) a
Spike dry weight (kg.ha^-1^)	2290 (12.6) a	2283 (33.4) a	–	2224 (17.1) a	4953 (58.3) b	2661 (41.5) a
Thousand-seed weight (g)	42.7 (2.9) a	41.1 (1.9) a	–	42.7 (2.9) a	53.9 (2.7) b	52.8 (4.5) b
Shoot N content (%)	nd^3^	nd^3^	–	5.08 (0.31) a	5.55 (0.17) b	4.71 (0.41) a
Shoot P content (g.kg^-1^)	nd^3^	nd^3^	–	6.01 (0.59) a	7.47 (0.38) b	7.56 (0.34) b
Mycorrhizal colonization (%)	46.7 (5.8) a	62.4 (6.4) b	–	54.7 (4.7) a	74.7 (4.8) b	69.9 (3.2) b
Hyphal length (m g^-1^ dry soil)	1.66 (0.08) a	2.85 (0.07) c	2.14 (0.06) b	1.72 (0.07) a	2.98 (0.05) b	2.85 (0.04) b
Catabolic evenness	15.2 (0.83) b	12.3 (0.23) b	17.4 (0.48) c	15.1 (1.13) b	12.7 (1.01) a	13.5 (1.23) ab
Carbohydrates	0.025 (0.01) a	0.029 (0.02) b	0.028 (0.02) b	0.024 (0.02) a	0.021 (0.01) a	0.023 (0.02) a
Amino-acids	0.008 (0.01) a	0.006 (0.01) a	0.008 (0.01) a	0.007 (0.01) a	0.005 (0.01) a	0.006 (0.01) a
Carboxylic acids	0.067 (0.04) a	0.102 (0.07) b	0.060 (0.03) a	0.064 (0.04) a	0.075 (0.05) b	0.078 (0.05) b

*^1^Standard error. ^2^For each year, data in the same line followed by the same letter are not significantly different according to the Newman–Keul’s test (*p* < 0.05). ^3^nd, not determined.*

### Mycorrhizal Soil Infectivity and Soil Microbial Functionalities

The mycorrhizal colonization of wheat root systems was significantly higher in the WF treatment compared to the wheat monoculture after one cropping season (**Table 1**). After two cropping seasons, wheat root systems were highly colonized by the native mycorrhizal fungi in the WF and W+F treatments compared to the wheat monoculture (**Table 1**). The development of the mycorrhizal hyphal network was also stimulated in the intercropping and rotation treatments with highest values recorded in the WF treatment after the first growing season (**Table 1**).

In 2012, the catabolic evenness ranged as follow W+F > W = WF whereas it ranged as W > W+F > WF in 2013 (**Table 1**). No significant differences were recorded for the average amino-acid induced respiration within the treatments in 2012 and 2013 and for the average carbohydrate induced respiration in 2013 (**Table 1**). In contrast, the average SIR was significantly higher with carbohydrates in the WF and W+F treatments after the first growing season (**Table 1**). For the carboxylic acids, highest values were measured in 2012 with the WF treatment and in 2013 with the WF and W+F treatments (**Table 1**).

**FIGURE 1:**
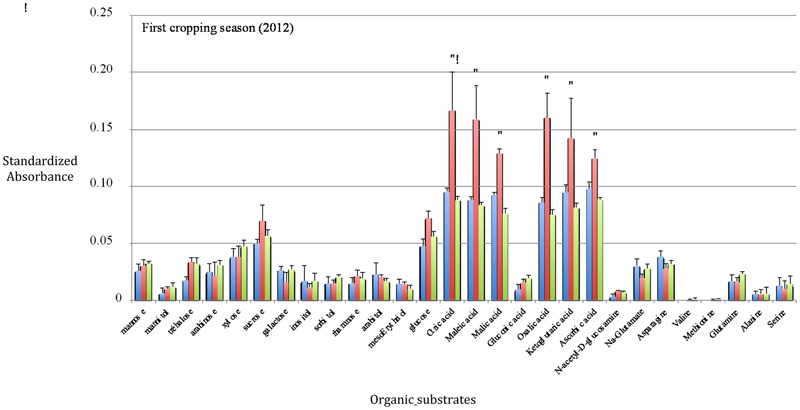
***In situ* catabolic potential (ISCP) profiles after the first growing season (2011–2012).** Error bars represent standard errors (*n* = 4). Blue bars: wheat monoculture (W); Red bars: wheat/Faba bean intercropping (WF); Green bars: Faba bean/wheat rotation (W+F). An asterisk indicates a significant difference between the WF treatment and the others according to the Newman–Keul’s test (*P* < 0.05).

**FIGURE 2:**
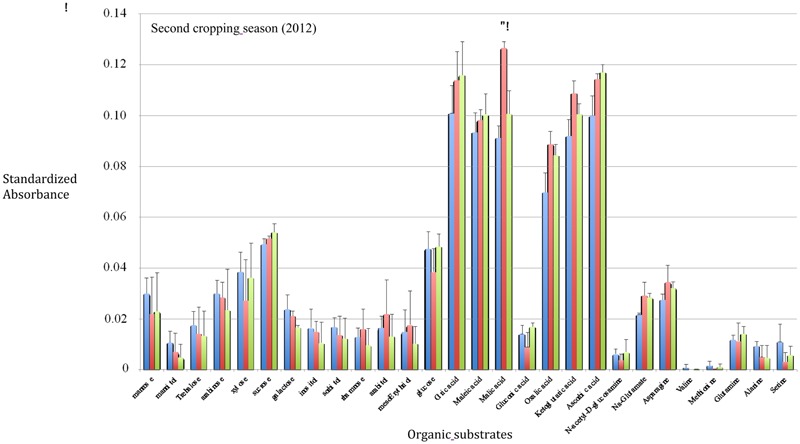
***In situ* catabolic potential profiles after the second growing season (2012–2013).** Error bars represent standard errors (*n* = 4). Blue bars: wheat monoculture (W); Red bars: wheat/Faba bean intercropping (WF); Green bars: Faba bean/wheat rotation (W+F). An asterisk indicates a significant difference between the WF treatment and the others according to the Newman–Keul’s test (*P* < 0.05).

**FIGURE 3:**
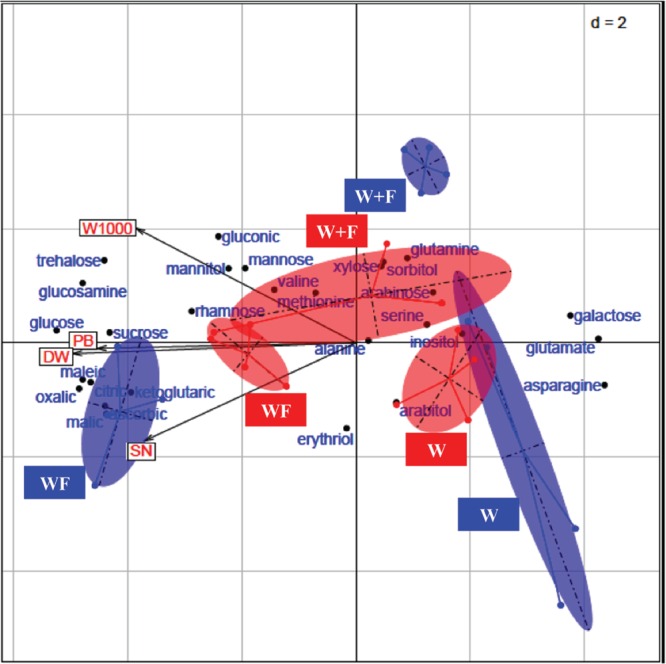
**Between-Group CO-Inertia Analysis (BGCOIA) on patterns of ISCP profiles (2012) and yield variables (2013).** The three red ellipses represent the samples of the yield variables table in 2013 for the three treatments (W = wheat monoculture, W+F = wheat/Faba bean rotation, WF = wheat/Faba bean intercropping). The four yield variables (in red) are represented by the four arrows pointing to the left (W1000, weight of 1000 grains, PB, plant biomass, DW, dry weight of spikes, SN, spike number). The three blue ellipses represent the samples of the ISCP profiles table in 2012 for the same three cultivation modes. The names of the organic substrates are given in blue.

After one cropping season, the highest SIRs have been obtained with citric acid, maleic acid, malic acid, oxalic acid, ketoglutaric acid, and ascorbic acid in the WF treatment (**Figure 1**) whereas and after two cropping seasons, a significant highest SIR response was only recorded with maleic acid in the WF treatment (**Figure 2**). The BGA permutation test of ISCP profiles in the three treatments (W, W+F, WF) is highly significant (*p* ∼1.10 10^-4^) in 2012, but not in 2013. The BGA permutation test for the comparison of SIR profiles in 2012 vs. 2013 is also significant (*p* ∼0.03). There is no relationship between SIR profiles and yield variables in 2012 or in 2013 (the permutation tests of Co-Inertia Analyses are not significant). But there is a strong relationship between 2012 SIR profiles and 2013 yield variables (*p* ∼0.004; **Figure 3**). Samples with higher yields are on the left, they correspond to intercropping (WF), and to high consumption of the following organic substrates: trehalose, glucosamine, glucose, sucrose, and organic acids (**Figure 3**). Lower yield levels are on the right, they correspond to wheat monoculture (W), and to the following organic substrates: galactose, glutamate, asparagine (**Figure 3**). On the vertical axis, rotation system (W+F) correspond to intermediate yield levels, and to particular organic substrates: gluconic acid, glutamine, xylose, mannose, mannitol, sorbitol (**Figure 3**).

## Discussion

This study conducted during two growing seasons clearly shows that intercropping could lead to highest performances in terms of crop yield than the rotation and the monoculture after two cropping seasons. Benefits resulting from intercrop seem to be linked with changes recorded on the mycorrhizal soil infectivity and on soil microbial functionalities.

These results are in accordance with previous studies showing that productivity advantages of intercropping may arise from complement use of growth resources such as N and water in either space or time (Akter et al., 2004; Chu et al., 2004). In the same way, intercropping legume and cereals may lead in highest nitrogen content in the cereal grain, improving that quality criterion (Bulson et al., 1997; Gooding et al., 2007).

The importance of above-belowground interactions in both natural and agricultural systems has been highlighted during the last decade (Wolters et al., 2000; Soler et al., 2012; Orrell and Bennett, 2013). Mixing plant species will create new habitats for associated species and more particularly through its impact on the soil microbiota composition (Bartelt-Ryser et al., 2005). In particular changes in plant cover composition alter the composition of Arbuscular Mycorrhizal (AM) fungal communities (Lovelock et al., 2003). AM fungi facilitate plant uptake and transport of less mobile soil nutrients (Jakobsen et al., 2001), enhance drought tolerance (Kaya et al., 2003) and reduce pathogenic infections (Abdallah and Abdel-Fattah, 2000). These fungal symbionts are also involved in the biological mechanisms that influence plant community productivity and plant-plant interactions (Wagg et al., 2011). It has been also reported that an AM fungal diversity increase could relax competition in species network (Bastolla et al., 2009). Although AM fungi have been traditionally believed to be non-host specific in their ability to infect and to promote the host plant growth, the benefits expected from the mycorrhizal symbiosis to enhance the plant host development may highly depend on the particular species involved (Bever et al., 2001; Jansa et al., 2005). For instance, it is well known that plant species, highly dependent to the mycorrhizal symbiosis for their growth (i.e., legumes) will promote the development of the mycorrhizal fungal growth, spore production, and hyphal network extend (Duponnois et al., 2001). Our results corroborate these previous studies as higher plant development; better mineral nutrition and higher mycorrhizal development were recorded in the intercropping treatment after one growing season.

Interactions of AM fungi with soil microbiota are an important driver of plant growth (Linderman, 1988; Johansson et al., 2004; Artursson et al., 2006). AM symbiosis is known to promote root exudation (Rambelli, 1973), and to influence rhizosphere microbial communities (Johansson et al., 2004). All these interactions constitute a plant root-mycorrhiza-bacteria continuum named “mycorrhizosphere.” In the current study, the soil compartment can be assimilated to the mycorrhizosphere. After one growing season, soil microbial functionalities were highly impacted by the plant cover composition (monoculture vs. intercropping) highlighting the importance of these aboveground-belowground interactions. The main differences were observed between the treatments with SIR response to carboxylic acids recorded with the intercropping treatment. AM fungi and their associated bacteria may excrete carboxylic acids as well as faba bean (Zhou et al., 2009; Hafidi et al., 2013). These organic compounds could also exert a selective influence on soil microbial communities by enhancing the multiplication of microorganisms able to catabolise organic acids (Ouahmane et al., 2007; Hafidi et al., 2013).

In the current study, a strong relationship between 2012 SIR profiles and 2013 yield variables was recorded. This result showed that intercropping strongly impacted soil microbial functionalities resulting in a positive effect of this cultural practice recorded again in the second cropping year.

## Conclusion

This field experiment demonstrates that the benefits of intercropping are highly subjected to the mycorrhizal symbiosis establishment and its impact on the soil microflora functionalities. In the present study, intercropping gave better results than the rotation practice in terms of crop productivity. However, this study has been only performed during two growing years and other studies have to be undertaken for more than two growing seasons to evaluate the long-term impact of intercropping compared to that resulting from rotations. These results emphasize the need to develop crop diversity in agroecosystems and to include the management of AM fungal communities in agro-ecological strategies in order to sustainably maintain the crop productivity. Another point of major interest would be to check, in different situations and over several crop rotations, the predictive validity of the SIR approaches as a bioindicator of soil high productivity with wheat and other cereals. In other words, in the absence of any chemical inputs, are SIR values valid indicators of soil resilience, across several successive legume/crops associations? Limiting SIR substrates to carboxylic acids would be a way to simplify and reduce the cost of such a biotest, as to maintain its reliability.

## Author Contributions

SW, YP, JT, HS, EB, and RD: These authors contributed to defining the objectives of the experiment, the interpretation of results, and the redaction of the article. TM, KO, CL, AG, and MH: These authors contributed to the implementation of the experiences and proofreading of the article.

## Conflict of Interest Statement

The authors declare that the research was conducted in the absence of any commercial or financial relationships that could be construed as a potential conflict of interest.
